# Inheritance

**Published:** 1883-05

**Authors:** 


### INHERITANCE.

WHEN a boy, a gentleman had the skin of both thumbs badly cracked from exposure to cold, combined with some skin disease. His thumbs swelled greatly, and remained in this state for a long time. When they healed they were misshapen, and the nails ever afterward were singularly narrow, short, and thick. This gentleman had four children, of whom the eldest, Sarah, had both her thumbs and nails like her father's ; the third child, also a daughter, had one thumb similary deformed. The two other children, a boy and girl, were normal. The daughter Sarah had four children, of whom the eldest and the third, both daughters, had their two thumbs deformed ; the other two children, a boy and girl, were normal. The great-grandchildren of this gentleman were all normal. Mr. Bishop believes that the old gentleman was correct in attributing the state of his thumbs to cold, aided by skin disease, as he positively asserted that his thumbs were not originally misshapen, and there was nor ecord of any previous inherited tendency of the kind in his family. He had six brothers and sisters, who lived to have families, some of them very large families- and in none was there any trace of deformity in their thumbs.

Several more or less closely analogous cases have been recorded ; but, until within a recent period, every one naturally felt much doubt whether the effects of a mutilation or injury were ever really inherited, as accidental coincidences would almost certainly occasion, ally occur. The subject, however, now wears a totally different aspect, since Dr. Brown-Séquard's famous experiments, proving that guinea-pigs of the next generation were affected by operations on certain nerves. Mr. Eugene Dupuy, of San Francisco, California, has likewise found, as he informs me, that with these animals, "lesions of nerve-trunks are almost invariably transmitted." For instance, "the effects of sections of the cervical sympathetic on the eyes are reproduced in the young, also epilepsy (as described by my eminent friend and master, Dr. Brown-Séquard) when induced by lesions of the sciatic nerve." Mr. Dupuy has communicated to me a still more remarkable case of the transmitted effect on the brain from any injury to a nerve ; but I do not feel at liberty to give this case, as Mr. Dupuy intends to pursue his researches, and will, as I hope, publish the results.—*Darwin.*

